# Survival processing modulates the neurocognitive mechanisms of episodic encoding

**DOI:** 10.3758/s13415-020-00798-1

**Published:** 2020-05-19

**Authors:** Glen Forester, Meike Kroneisen, Edgar Erdfelder, Siri-Maria Kamp

**Affiliations:** 1 Department of Psychology, University of Trier, Johanniterufer 15, 54290 Trier, Germany; 2 University of Koblenz-Landau, Mainz, Germany; 3 University of Mannheim, Mannheim, Germany

**Keywords:** Episodic memory, ERP, Motivation, Survival processing, Subsequent memory effects

## Abstract

Memories formed in the context of an imagined survival scenario are more easily remembered, but the mechanisms underlying this effect are still under debate. We investigated the neurocognitive processes underlying the survival processing effect by examining event-related potentials (ERPs) during memory encoding. Participants imagined being either stranded in a foreign land and needing to survive, or in an overseas moving (control) scenario, while incidentally encoding a list of words. Words encountered in the survival context were associated with improved recall and reduced false-memory intrusions during a later memory test. Survival processing was associated with an increased frontal slow wave, while there was no effect on the overall P300 amplitude, relative to the control scenario. Furthermore, a subsequent memory effect in the P300 time window was found only in the control scenario. These findings suggest that survival processing leads to a shift away from lower level encoding processes, which are sensitive to motivation and stimulus salience and which were evident in the control scenario, to more active and elaborative forms of encoding. The results are consistent with a richness of encoding account of the survival processing effect and offer novel insights into the encoding processes that lead to enhanced memory for fitness-relevant information.

## Introduction

The human memory system has presumably been shaped according to its effects on evolutionary fitness and therefore may be “tuned” to preferentially process fitness-relevant information (Nairne & Pandeirada, 2016). However, the proximate mechanisms by which such fitness-relevant information gains an advantage in episodic memory remain controversial. The issue has garnered increasing attention because of its implications for understanding both the functions of memory as evolutionary adaptations, as well as the processes through which these functions are achieved (Schwartz, Howe, Toglia, & Otgaar, 2014). Event-related potentials (ERPs) provide a way of observing whether and how memory encoding processes are affected by fitness-relevance, and we thus employed ERPs to examine the contribution of motivation, salience, and elaborative encoding processes to memory enhancement for fitness-relevant information.

### The survival processing effect

Nairne, Thompson, and Pandeirada (2007) first tested the hypothesis that fitness-relevant information is preferentially processed by prompting participants to imagine themselves in either a survival scenario, in which they were stranded in foreign grasslands without material resources, or in an overseas moving (control) scenario and found that information encountered in the survival context was better remembered during a later memory test. This “survival processing effect” has been found against a variety of different control scenarios and other highly effective mnemonic-enhancement techniques (for recent reviews see Kroneisen & Erdfelder, 2017; Nairne & Pandeirada, 2016). However, the mechanisms underlying the effect remain controversial (Erdfelder & Kroneisen, 2014). For example, some researchers have suggested that the effect may be due to motivation or emotional arousal induced by the survival context (Fiacconi, Dekraker, & Köhler, 2015; Kang, McDermott, & Cohen, 2008; Otgaar, Smeets, & van Bergen, 2010), whereas others have proposed that items may be more relevant or semantically congruent to the survival scenario (Butler, Kang, & Roediger, 2009; Palmore, Garcia, Bacon, Johnson, & Kelemen, 2011). Furthermore, some have offered that gist or schema processing might play a crucial role, an explanation supported by the finding that survival processing boosts not only correct recall but apparently also incorrect recall (i.e., intrusions) if item lists are semantically homogenous (Otgaar & Smeets, 2010; Howe & Derbish, 2010).

Still other explanations focus on the nature of single-item encoding as the crucial mechanism driving the survival processing advantage. Specifically, the richness of encoding hypothesis posits that the survival processing effect is due to richer elaboration at the time of encoding, as the survival context fosters more and more varied possibilities for evaluating possible functions of objects in the respective context (Bell, Röer, & Buchner, 2015; Kroneisen & Erdfelder, 2011; Kroneisen, Erdfelder, & Buchner, 2013; Röer, Bell, & Buchner, 2013). Support for this hypothesis has come from several lines of evidence. For example, the survival processing effect is abolished when to-be-remembered words are low in imageability or concreteness (Bell, Röer, & Buchner, 2013; Kroneisen & Makerud, 2017; Forester, Kroneisen, Erdfelder, & Kamp, 2019), the complexity of the survival scenario is limited (Kroneisen & Erdfelder, 2011; but see Klein 2013), or the cognitive resources available for elaboration are reduced by means of a secondary task (Kroneisen, Rummel, & Erdfelder, 2014, 2016). These patterns can be attributed to the fact that abstract words, less complex scenarios, or situations with reduced processing resources do not provide the same capacity for elaboration on the functional uses of an item as do the standard survival processing conditions employed by Nairne et al. (2007). Furthermore, we recently found that survival processing increases both behavioral and electrophysiological measures of recollection during memory retrieval (Forester et al. 2019). Recollection involves bringing to mind rich details associated with a memory, and it is sensitive to increases in elaboration at the time of encoding (Yonelinas, 2002).

One limitation to these prior studies is that they have relied on indirect inference about encoding processes derived from performance (or brain activity) on later memory tests. Consequently, it is difficult to tease apart the influence of survival processing on genuine encoding processes from ensuing effects on memory storage, consolidation, or retrieval. In order to test the richness of encoding hypothesis against competing hypotheses while examining encoding processes directly, the present study recorded ERPs during the encoding phase of a survival-processing paradigm.

### ERPs as measures of encoding mechanisms

ERPs are real-time measures of neural activity recorded from the scalp. Different ERP components with characteristic time courses and scalp distributions reflect distinct neurocognitive (sub-) processes, allowing for mechanistic insights into processes, such as episodic memory encoding. Furthermore, the high temporal resolution of ERPs allows for a dissociation of processes indexing memory encoding from subsequent consolidation and retrieval. Different ERP components have been shown to be relevant for successful episodic memory, and two that are particularly sensitive to memory encoding mechanisms are the P300 and the frontal slow wave.

The P300 is a parietal positivity that peaks at least 300 ms, but up to 700 ms, following an unexpected or otherwise salient stimulus (Sutton, Braren, Zubin, & John, 1965). The P300 is independent of, and occurs subsequent to, low-level sensory or perceptual processes. It is associated with the initial classification and encoding of stimulus meaning and is thought to index the updating of a mental representation of the environment in memory (Donchin, 1981). Furthermore, the P300 is sensitive to the cognitive resources allocated to stimulus categorization processing, because its amplitude is increased by tasks or stimuli that increase motivation or emotional arousal (for reviews, see Johnson, 1986; Hajcak, MacNamara, & Olvet, 2010).

Slow-wave ERPs are sustained ERP deflections with a later onset than the P300, which vary with working memory load (Ruchkin, Johnson, Canoune, & Ritter, 1990) and content (Mecklinger & Pfeifer, 1996). Hence, slow waves are assumed to represent active forms of maintenance and manipulation within working memory (Johnson, 1995). In particular, a frontally distributed slow wave reflects processes of executive control over information maintained in working memory (Bosch, Mecklinger, & Friederici, 2001). Slow waves are also commonly observed in episodic encoding tasks. Specifically, the frontal slow wave is more positive (or less negative) when encoding processes are more elaborative (Fabiani, Karis, & Donchin, 1990; Karis, Fabiani, & Donchin, 1984; Mecklinger & Müller, 1996). The amplitude of the frontal slow wave is associated with long-term memory formation for individual items (Khader, Ranganath, Seemüller, & Rösler, 2007) as well as for lists of items (Kamp, Lehman, Malmberg, & Donchin, 2016).

Due to the relatively well-characterized functional significance of the two ERP components, examining their sensitivity to the survival context can provide unique theoretical insights into the mechanisms underlying the survival processing effect. For example, if the survival processing effect is due to a general increase in resource allocation, caused by increased motivation or arousal (Fiacconi et al., 2015; Soderstrom & McCabe, 2011), then survival processing should be associated with an increased P300. In contrast, if survival processing increases working-memory-based elaboration, it should be associated with a more positive frontal slow wave.

One previous study has examined encoding ERP activity during survival processing (Zhang, Li, & Guo, 2020), and the results indicated that the survival scenario, compared with the moving scenario, was associated with an increased parietal positivity that is morphologically consistent with the P300. However, the authors did not examine ERPs beyond the P300 time window, making it difficult to separate initial word categorization processes from later, more active encoding processes. Furthermore, they did not investigate how differences at encoding were associated with subsequent memory for a given word, an issue that can be addressed using the subsequent memory paradigm.

### P300 and frontal slow wave subsequent memory effects

The subsequent memory paradigm investigates brain activity at encoding that is associated with successful subsequent memory retrieval on a trial-by-trial basis (Sanquist, Rohrbaugh, Syndulko, & Lindsley, 1980). Specifically, brain activity elicited by a sequence of items presented during the encoding phase of a memory experiment is back-sorted and averaged depending on performance on a subsequent memory test. When brain activity differs between subsequently recalled and subsequently unrecalled items, this is referred to as a subsequent memory effect (SME). Subsequent memory effects therefore reflect trial-to-trial variations in neurocognitive activity during encoding that systematically relate to subsequent retrieval success (Paller & Wagner, 2002). The P300 and the frontal slow wave are the ERP components most frequently reported to show SMEs, but the circumstances under which each SME is observed are dissociable.

An SME in the P300 time window is observed for items that are physically or semantically salient when the initial salience of items is the key predictor of successful memory retrieval (Fabiani, Karis, & Donchin, 1986; Karis, et al., 1984). As with the P300, this SME tends to have a centro-parietal maximum, but it often is more widely distributed across the scalp (Kamp, Bader, & Mecklinger 2017). However, for the sake of simplicity, and due to its co-occurrence with the P300, we will refer to this SME as the “P300 SME.” When elaborative processes are used during encoding, the P300 SME tends to disappear, presumably because the initial salience of an item becomes overshadowed as a retrieval cue by the products of elaboration (Fabiani, et al., 1990). For example, when items are encoded using elaborative, “deep” encoding (Karis et al., 1984; Fabiani et al., 1990; Guo, Zhu, Ding, Fan, & Paller, 2004; Liu, Rosburg, Gao, Weber, & Guo, 2017), or when items are encoded associatively as pairs (Kamp & Zimmer, 2015), a frontal slow wave SME, rather than the P300 SME, often is found. The frontal slow wave SME therefore may reflect increased working-memory-based elaboration for some items, which could include forming or manipulating associations between items, an item and its context, or an item and information from long-term memory—thus improving the memorability of those items (Kamp et al., 2017).

Therefore, if resource allocation due to the initial salience of some words is responsible for the survival processing effect, then the P300 SME should be larger in a survival scenario compared with a control scenario. Multiple factors could contribute to the salience of a word, but one factor of particular relevance is the semantic congruence of a word with the scenario (Butler et al., 2009). Words that stand out due to high congruence with the scenario should elicit a larger P300 than other words, a pattern that has indeed been reported previously (Zhang et al., 2020). Crucially, if heightened congruency (or any other mechanism related to salience or resource allocation) for subsets of words underlies the survival processing effect, despite similar congruency on average, then the amplitude of the P300 should be associated with subsequent memory on an item-by-item basis, producing a P300 SME. In contrast, if higher-level elaborative processes are more important for survival processing, then the P300 SME should be reduced or disappear. Furthermore, in the case of increased elaboration, the frontal slow wave and the frontal slow wave SME should be larger during survival processing, reflecting a stronger association between the amount of elaboration for a given word and its propensity for being recalled. Note that these SMEs can be independent of any potential scenario effects on the overall ERP amplitudes. For example, an increase in overall resource allocation could lead to a generally increased P300, and an increase in working memory-based elaboration could lead to an increased frontal slow wave, but these factors would only lead to SMEs if their word-by-word variation were associated with subsequent memory performance.

Using oscillatory EEG activity rather than ERP activity, one previous study has utilized the subsequent memory paradigm to investigate the survival processing effect (Fellner, Bäuml, & Hanslmayr, 2013). The authors found alpha and beta power SMEs in a semantic control condition, but not in the survival condition. Frontal alpha and beta power often reflects semantic processing during perception (Hanslmayr, Staresina, & Bowman, 2016), and alpha and beta power SMEs have been linked specifically to semantic (compared to nonsemantic) encoding (Hanslmayr, Spitzer, & Bauml, 2009). Thus, the lack of alpha and beta power SMEs during survival processing indicates that lower-level semantic processing was less important for memory formation in the survival condition. In addition, the authors observed alpha and beta long-range phase synchrony SMEs only in the survival condition. Long-range phase synchrony likely reflects communication between distant cortical areas (Varela, Lachaux, Rodriguez, & Martinerie, 2001), and enhanced cortico-cortical communication during survival processing could reflect the integration and elaboration of information across multiple domains (Fellner et al., 2013). These results are therefore consistent with predictions derived from the richness of encoding hypothesis of a P300 SME in the control condition only (indicating that initial word-categorization processes are closely associated with successful memory formation in typical word-encoding paradigms, but not during survival processing) and a frontal slow wave SME in the survival condition only (indicating that more active elaboration is key to memory for words processed in a survival context).

### Present study

In the present study, we manipulated survival processing between subjects, using the standard survival and moving (control) scenarios (Nairne et al., 2007), and recorded participants’ EEG during an incidental encoding task, followed by a free recall test. We then tested whether survival processing influenced neurocognitive (ERP) processes during encoding relative to the control condition. Specifically, we tested four hypotheses. First, if the survival processing effect is caused by an increase in motivation or arousal, then P300 amplitude should be larger in the survival group than in the control group. Alternatively, if motivation and arousal are unaffected by survival processing, as predicted by the richness of encoding hypothesis, then no group difference in P300 amplitude should be found. Second, if survival processing affects the initial salience of some words in the survival context, thereby enhancing memory encoding, then the P300 SME should be enhanced, but if it increases elaboration, as the richness of encoding hypothesis predicts, then the P300 SME should be equal or smaller in the survival group compared to the control group. Third, if, and only if, the effect is due to increased elaborative processes, then a more positive frontal slow wave should be observed in the survival group compared to the control group. Fourth, if survival processing enhances the relevance of elaboration to encoding on a word-by-word basis, then the frontal slow wave SME should be larger for the survival group than it is for the control group. However, if elaboration occurs on a block-wise basis (or not at all), then the frontal slow wave SME should not differ between groups.

## Methods

The local ethics committee at Trier University approved the study, and all procedures were performed according to the ethical standards of the German Psychological Association.

### Participants

Participants ranged in age from 18 to 31 years (mean [*M*] = 23.6, standard deviation [*SD*] = 2.81), and received either partial course credit or 8.50 Euros per hour for their participation. A total of 102 participants (29 males) took part in the study. Fifty-one participants were randomly assigned the survival scenario, and 51 were randomly assigned the moving scenario, yielding a one-factor between-subjects design. The number of participants was determined in advance using an a priori power analysis (G*Power; Faul, Erdfelder, Buchner, & Lang, 2009) to detect a medium-sized survival processing effect (*d* = 0.5) on behavioral recall performance (Scofield, Buchanan, & Kostic, 2017) using a one-tailed *t*-test, given an α of 0.05 and a desired power of 0.80.

### Stimuli

Sixty German nouns from the Berlin Affective Word List Reloaded (BAWL-R; Võ et al., 2009) were used as critical words for the study, plus an additional 12 used as practice and buffer words. These words represent a subset of the words used in a previous study (Forester et al., 2019). Because fewer words were needed in the present study, we excluded the words that had received the largest group differences in relevance ratings in our prior study to help control for overall differences in congruity (Butler et al., 2009). All words were high in imageability (Kroneisen & Makerud, 2017; Forester, et al., 2019) but were moderate with regard to valence, arousal, and frequency [imageability: *M* = 6.0, *SD* = 0.3, on a scale from 1 (hardly imageable) to 7 (very imageable); valence: *M* = 0.15, *SD* = 0.83, on a scale from −3 (very negative) to 3 (very positive); arousal: *M* = 2.6, *SD* = 0.53, on a scale from 1 (low-arousing) to 5 (high-arousing); frequency: *M* = 53.1 per million, *SD* = 142.6]. All words were between 4 and 8 letters in length (*M* = 6.23, *SD* = 1.15).

### Scenarios

We used the standard survival and moving scenarios, first introduced by Nairne et al. (2007), which we translated into German. The scenarios were as follows.

- Survival: *In this task, we would like you to imagine that you are stranded in the grasslands of a foreign land, without any basic survival materials. Over the next few months, you’ll need to find steady supplies of food and water and protect yourself from predators. We are going to show you a list of words, and we would like you to rate how relevant each of these words would be for you in this survival situation. Some of the words may be relevant and others may not—it’s up to you to decide.*
- Moving: *In this task, we would like you to imagine that you are planning to move to a new home in a foreign land. Over the next few months, you’ll need to locate and purchase a new home and transport your belongings. We are going to show you a list of words, and we would like you to rate how relevant each of these words would be for you in accomplishing this task. Some of the words may be relevant and others may not—it’s up to you to decide.*

### Procedure

Before beginning the experiment, participants provided informed consent and the EEG recording preparation was completed. Participants then read either the survival or moving scenario, and completed six practice trials. Following the practice trials, participants were given the opportunity to ask the experimenter questions. The incidental encoding task then began, in which participants rated 60 words based on each word’s relevance to the scenario. This incidental encoding task was identical to the one used in Forester et al. (2019). The words were presented in random order, and participants rated each word on a scale from 1 (not at all relevant) to 5 (extremely relevant). Six buffer words, three at the beginning and three at the end of the task, were additionally included to absorb primacy and recency effects. Results from the buffer words were not included in the analyses. A self-paced break was provided halfway through the encoding task. All stimuli were presented in size 40, Courier New font on a gray background.

**Fig. 1.**
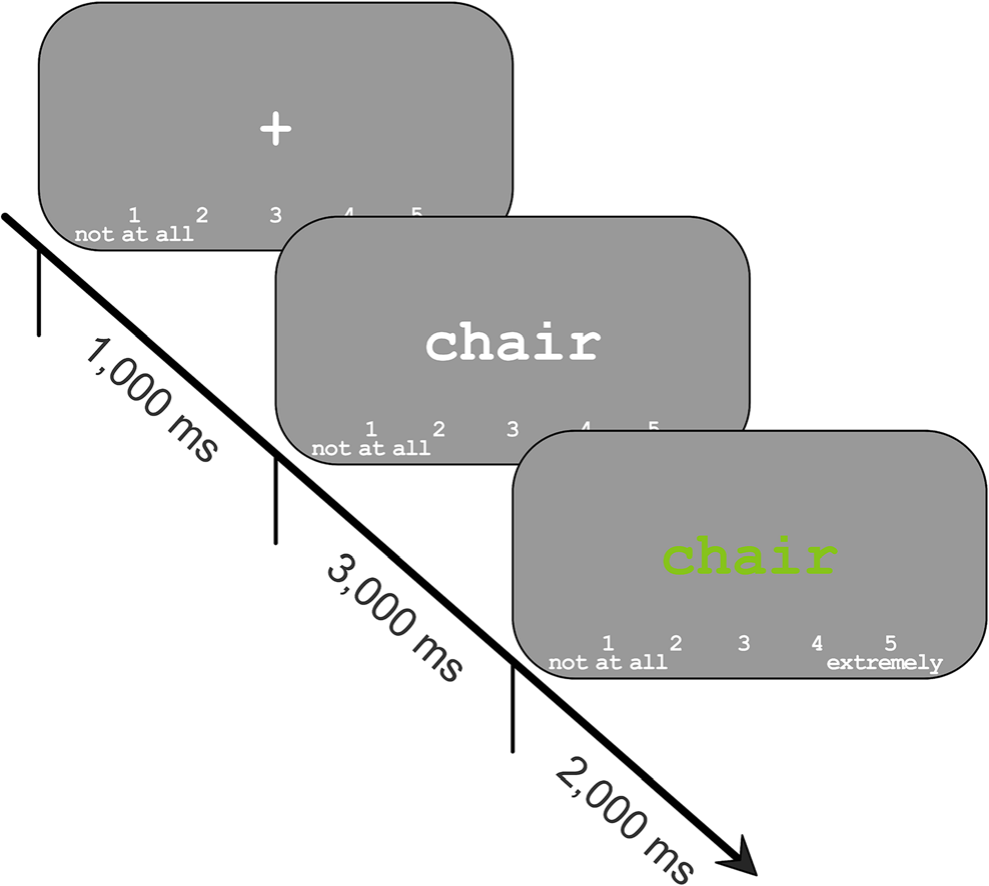
Incidental encoding task. Participants rated the relevance of each word to either a survival or a moving (control) scenario using a 5-point scale from “not at all” to “extremely” relevant. Each word was presented for a total of five seconds, and participants were instructed to provide the relevance rating during the final 2 seconds, while the word was green

A representation of an encoding trial is shown in Fig. 1. Each trial began with a fixation cross presented for 1,000 ms, which was then replaced by the to-be-rated word at the center of the screen. The word was presented for a total of 5,000 ms; for the first 3,000 ms, the word was displayed in white font, and for the remaining 2,000 ms, the word was displayed in green font. Participants were instructed to provide the relevance rating using a key press while the word was green. The rating scale was continuously visible at the bottom of the screen.

After all words were presented, participants completed an unrelated questionnaire for 3 minutes, which served as a distraction before the surprise recall test. For the recall test, participants were given 15 minutes to recall as many words as possible from the encoding task. Note that this recall time is somewhat longer than the typical 10 minute recall phase (Nairne et al., 2007), because twice the typical number of words were used at encoding. Participants typed the recalled words using the computer keyboard and were able to view and edit each word before submitting it. Once a word was submitted, it could no longer be edited, but all of the submitted words were continuously visible on the screen throughout the test. After 15 minutes had passed, the test ended and the participants were debriefed.

### Behavioral data analysis

A word submitted during the free recall test was counted as correctly recalled if it (1) exactly matched one of the 60 critical words during the encoding task, (2) was deemed an insignificant misspelling of a critical word by two independent scorers, or (3) was deemed an unimportant variation of a critical word (e.g., the plural form of singular critical word) by two independent scorers. A submitted word was counted as an intrusion if not counted as correctly recalled and did not match (and was not deemed an insignificant misspelling or variation of) a practice or buffer word presented during the encoding task.

### EEG recording and analysis

We recorded participants’ EEG using 33 Ag/AgCL electrodes, with the ground electrode at AFz, according to the extended 10/20 system. Using a NeuroOne amplifier (Bittium Corporation, Finland), the EEG was amplified from 0.16 to 7,000 Hz with an analogue 125-Hz low-pass filter and digitized at a rate of 500 Hz. Offline, using BrainVision Analyzer 2.0, the EEG was re-referenced from FCz to linked mastoids, and the signal at FCz was mathematically reconstructed. The data were then filtered using a 30-Hz low-pass IIR Butterworth filter with a 24 dB/octave roll-off and a 50-Hz notch IIR Butterworth filter with a 72 dB/octave roll-off. The EEG was segmented from 500 ms before word onset, which served as the baseline period, to 3,000 ms after word onset. The semiautomatic, infomax (Bell & Sejnowski, 1995) independent component analysis algorithm implemented in the BrainVision Analyzer was used to correct for ocular artifacts. Components detected by the procedure were manually screened, and only components that had clear eye-blink or saccade spatial and temporal distributions were rejected as ocular eye artifacts. Automatic artifact detection was then done to remove segments that contained an absolute amplitude difference of more than 30 μV in a time window of 1 ms or an absolute amplitude difference of more than 120 μV between the maximum and the minimum μV-value within the segment. Artifact-free EEG segments were averaged separately for each participant and separately for subsequently recalled and subsequently unrecalled words. Any participant who had fewer than 10 artifact-free trials included in an ERP average (i.e., <10 artifact-free trials elicited by subsequently recalled words or by subsequently unrecalled words) was excluded from the ERP analysis. As a result, 9 participants had to be excluded, leaving 46 participants in the survival group and 47 in the moving group. An average of 28 trials in the survival group and 24 trials in the moving group were included per subject-ERP average for subsequently recalled words, and an average of 29 trials in the survival group and 34 trials in the moving group for subsequently unrecalled words. Finally, the ERP averages were baseline corrected using the 500-ms baseline period.

To investigate the group and subsequent memory ERP effects, we analyzed ERPs within two time windows, corresponding to the P300 (500–700 ms) and frontal slow wave (1,000–2,000 ms), respectively, as well as SMEs in the corresponding time windows. These time windows were selected, because they correspond to the typical timing of maximum P300 (Fabiani et al., 1990; Kamp et al., 2016) and frontal slow wave (Kamp et al., 2017; Kamp, Endemann, Domes, & Mecklinger, 2019) effects when using similar designs, and the suitability of the time windows was confirmed by visual inspection of the present data. Within each time window, we measured mean ERP amplitude at nine electrodes (F3, Fz, F4, C3, Cz, C4, P3, Pz, and P4), forming a 3 (Anteriority) by 3 (Laterality) electrode grid. These electrodes were chosen to cover both components of interest, based on the typical centro-parietal distribution of the P300 (Spencer, Dien, & Donchin, 2001) and the typical fronto-central distribution of the frontal slow wave (Bosch, et al., 2001; Kamp, et al., 2019).

### Statistical analysis

To test for the typical survival processing recall advantage, we compared the proportion of correctly recalled words between the survival and moving groups using a one-tailed, between-subjects *t*-test. Further, using two-tailed, between-subjects *t*-tests, we compared the number of intrusions between groups to test for differences in false memory, and we compared the mean relevance ratings from the encoding task between groups to determine if the words were rated as equally relevant to the two scenarios. Note that all 102 participants were included in the behavioral analyses, including the nine who were excluded from the ERP analysis, in order to retain power for detecting behavioral effects.

The mean ERP amplitude within each time window was compared statistically using 2 x 2 x 3 x 3 (Scenario x Subsequent Memory x Anteriority x Laterality) mixed factor ANOVAs, with Scenario as the between-subjects factor. The factor Scenario captures the group effects on the encoding ERP amplitudes, whereas the factor Subsequent Memory captures the SMEs by comparing the encoding ERPs elicited by subsequently recalled to subsequently unrecalled words. The electrode factors (Anteriority and Laterality) were included to allow for exploring potential differences in scalp distributions. However, to avoid alpha-error accumulation, effects of the electrode factors were reported only if they qualified significant main effects of Scenario or Subsequent Memory, or a significant interaction of Scenario with Subsequent Memory. We applied a Greenhouse-Geisser correction for any *F-*test that included at least one of the electrode factors (Jennings, 1987) and report the corrected degrees of freedom.

## Results

### Behavioral results

**Fig. 2.**
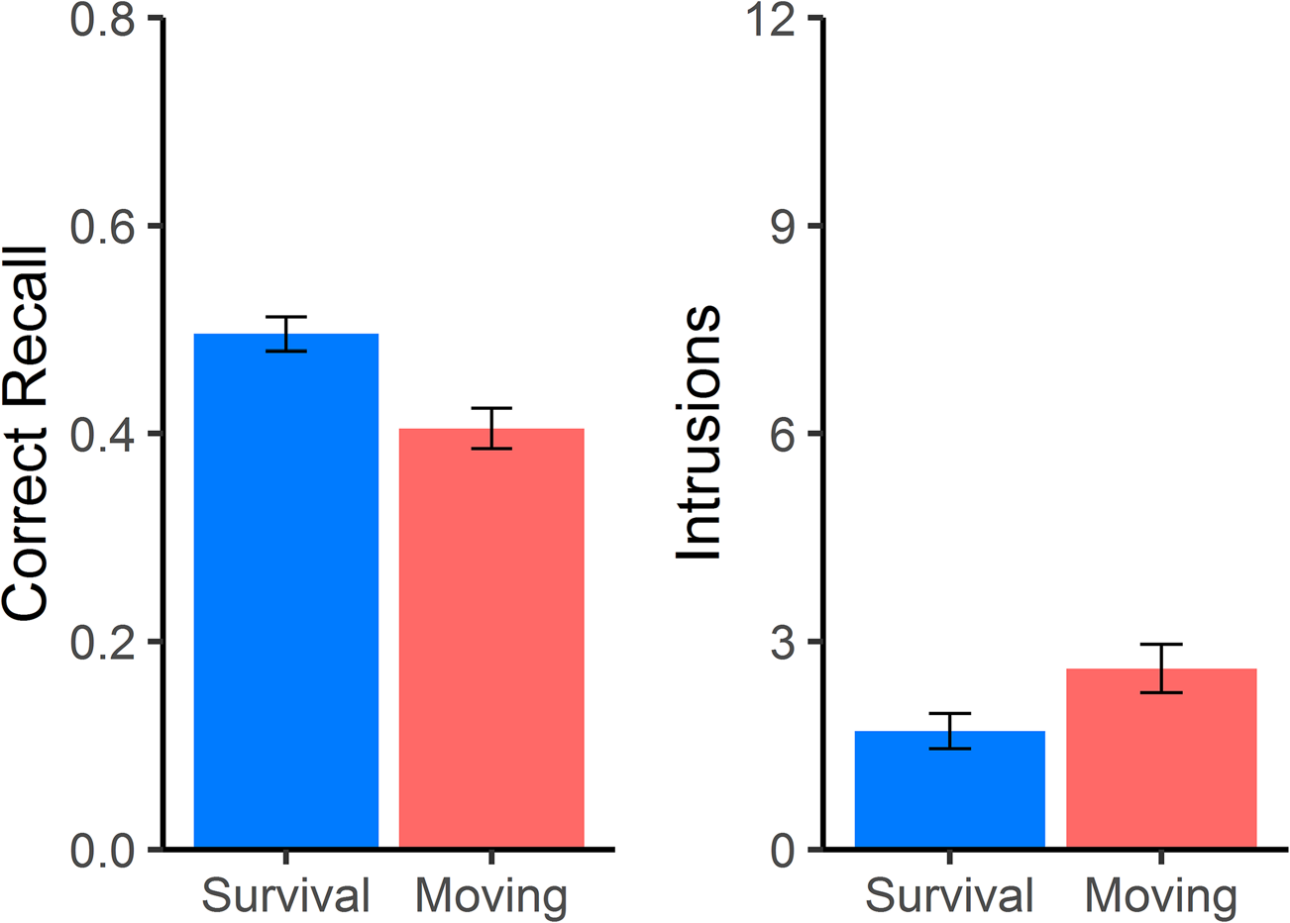
Behavioral results. Proportion of correctly recalled words (left side) and the number of intrusions (right side) during the free recall test. Error bars indicate standard errors of the mean

The mean proportions of correctly recalled words and the mean numbers of intrusions are shown in Fig. 2. Replicating the survival processing effect, significantly more words were recalled when encoded in the context of the survival scenario, *t*(100) = 3.58, *p* < 0.001, *d* = 0.71. There also was a significant difference in the number of intrusions between the groups, such that the survival scenario was associated with fewer falsely recalled words, *t*(100) = 2.09, *p* = 0.04, *d* = 0.41. The mean relevance ratings at encoding did not significantly differ between the survival (*M* = 2.88, *SD* = 0.46) and moving (*M* = 2.89 *SD* = 0.36) scenarios, *t*(100) = 0.13, *p* = 0.9, *d* = 0.03, indicating that the memory performance effects were not due to differences in the overall congruity of the words to the scenarios (Butler et al., 2009).

### ERP results

**Fig. 3.**
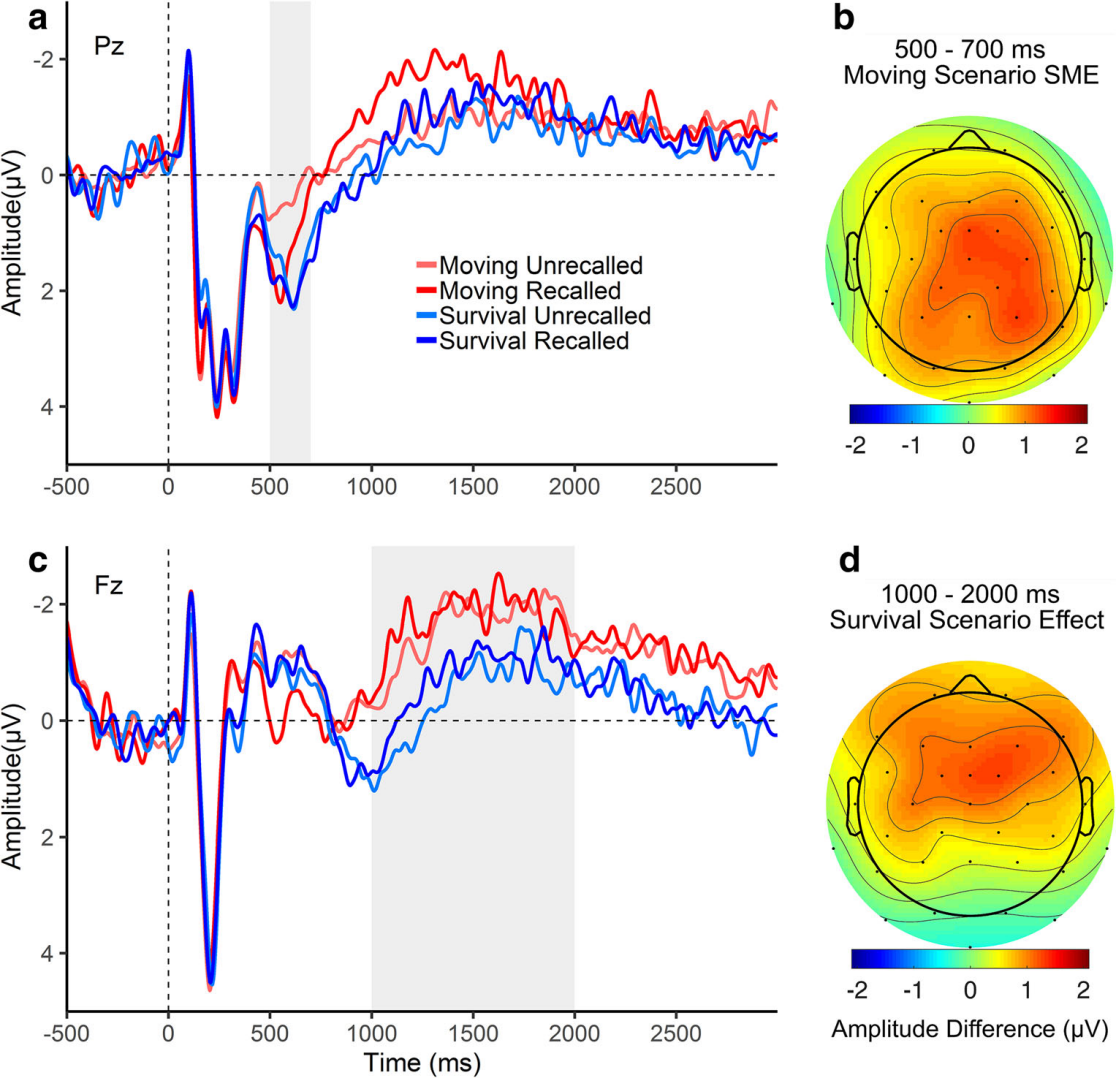
ERPs at two representative electrodes (A and C) elicited by words at encoding, and scalp distributions (B and D) of the statistically significant ERP effects. (**A**) ERPs at the midline-parietal electrode Pz. (**B**) Scalp distribution of the subsequent memory effect (SME; recalled – unrecalled words) in the moving (control) group during the P300 time window. (**C**) Encoding ERPs at the midline-frontal electrode Fz. (**D**) Scalp distribution of the amplitude difference between groups (survival – moving) in the frontal slow wave time window

Words presented during the encoding task elicited a positive peak, maximal at parietal electrodes and peaking at approximately 600 ms, consistent with the P300 (Fig. 3). This was followed by a sustained negative deflection in the ERPs, maximal at frontal electrodes, consistent with the frontal slow wave.

#### P300 time window (500–700 ms)

Statistical analysis of the P300 time window revealed a significant main effect of Subsequent Memory, *F*(1, 91) = 9.41, *p* = 0.003, η^2^_p_ = 0.09, such that at the time of encoding, subsequently recalled words elicited a greater positivity compared with subsequently unrecalled words. Significant Subsequent Memory x Laterality, *F*(1.93, 175.47) = 5.93, *p* = 0.004, η^2^_p_ = 0.06, and Subsequent Memory x Anteriority x Laterality, *F*(3.77, 342.64) = 2.96, *p* = 0.022, η^2^_p_ = 0.03, interactions reflected that the SME had a centro-parietal, right-lateralized maximum (see Fig. 3b for the distribution of the SME in the moving group).

The main effect of Scenario was not significant, *F*(1, 91) = 0.12, *p* = 0.725, η^2^_p_ = 0.00. However, there was a significant Scenario x Subsequent Memory interaction, *F*(1, 91) = 6.23, *p* = 0.014, η^2^_p_ = 0.06. The SME averaged across all 9 electrodes was only evident in the moving group, *t*(46) = 3.45, *p* = 0.001, *d* = 0.5, and not in the survival group, *t*(45) = 0.49, *p* = 0.627, *d* = 0.07.

#### Frontal slow wave time window (1,000–2,000 ms)

A main effect of Scenario, *F*(1, 91) = 8.26, *p* = 0.005, η^2^_p_ = 0.08, revealed that words in the survival group elicited a more positive slow wave than words in the moving group (Fig. 3d). There was neither a main effect of Subsequent Memory, *F*(1, 91) = 0.78, *p* = 0.381, η^2^_p_ = 0.01, nor a Scenario x Subsequent Memory interaction, *F*(1, 91) = 0.02, *p* = 0.886, η^2^_p_ = 0.00.

## Discussion

Replicating many prior findings, participants who evaluated words in the context of an imagined survival scenario demonstrated enhanced performance on a later memory test. This memory enhancement was associated with a distinct pattern of effects on encoding-related ERP components, providing strong evidence that survival processing affects the neurocognitive mechanisms of episodic encoding, and offering insight into a precise mechanistic view of the manner in which it does so. Specifically, with relation to the four hypotheses put forward in the introduction, we found (1) no difference in the overall P300 amplitude between groups, (2) a P300 SME in the moving (control) group only, (3) an enhanced frontal slow wave in the survival group, and (4) a frontal slow wave SME in neither group. These results indicate (1) that differences in motivation do not drive the survival processing effect; (2) that lower-level semantic processes are less important during survival processing; (3) that elaborative processes are increased during survival processing; and (4) that word-by-word variability in elaboration does not predict subsequent recall in either the survival or a control scenario. These findings largely confirm predictions derived from the richness of encoding hypothesis but are inconsistent with views that assume a key role of enhanced resource allocation, such as due to increased motivation or semantic salience, in underlying the survival processing effect. In the following sections, we will further examine the implications of these ERP findings, initially from the P300 and then from the frontal slow wave time window, for understanding the mechanisms underlying the effect. We will then address how our behavioral results add to the existing literature on the survival processing effect.

### No role of motivation and salience in the survival processing effect: evidence from P300 and the P300 SME

If survival processing leads to an increase in resource allocation because of increased motivation or arousal, larger P300 amplitudes should be elicited in the survival group (Hajcak et al., 2010; Johnson, 1986). We, however, found no significant group differences in the overall amplitude of the P300, which is consistent with the richness of encoding hypothesis and with many previous behavioral studies (Erdfelder & Kroneisen, 2014) in suggesting that a general increase in motivation or arousal is unlikely to be the primary factor driving the survival processing effect.^1^

It should be noted, however, that a recent study did report increased positivity associated with survival processing in the P300 time range (Zhang et al., 2020). This inconsistency may lie in the fact that in the study by Zhang et al. (2020), words were on average rated as more relevant in the survival scenario compared to the moving scenario, a difference that likely influenced their P300 effect. However, in the light of a descriptive pattern for an increased P300 in the present study, additional research is clearly needed to completely rule out a potential role of motivation, arousal, and resource allocation (reflected in the P300), in the survival-processing paradigm.

We further hypothesized that if the initial salience of some words, such as due to their semantic congruence with the scenario, were crucial to the survival processing memory advantage, one would expect a larger P300 SME in the survival group (Karis et al., 1984). Instead, we found an SME during the P300 time window in the moving group only. This SME in the moving group indicates that relatively early encoding processes are relevant for successful encoding of individual words in the present word-encoding paradigm, while the absence of a P300 SME in the survival group suggests that survival processing promotes a shift away from this—presumably less effective, lower-level—type of semantic encoding, towards more elaborative forms of encoding (see next section). This is consistent with the richness of encoding hypothesis and with results from a previous oscillatory EEG SME study (Fellner et al., 2013). Fellner et al. (2013) found that alpha and beta power, reflecting the amount of (low-level) semantic processing during encoding, only predicted subsequent memory in a control condition, and not in the survival condition.

It is worth noticing that the group difference in the P300 SME appears to be driven by a smaller P300 for unrecalled words in the moving relative to the survival group (one-tailed *t*-test on P300 amplitude averaged over the 3 parietal electrodes, *p* = 0.036), whereas there is no group difference for recalled words (*p* = 0.345; Fig. 3a). This could suggest that a homogeneously high level of cognitive resources was allocated to processing all words in the survival context, while only salient words recruited the same high level of resources in the moving context, selectively promoting their encoding success. This conclusion, at first glance, conflicts with a result pattern in our previous study (Forester et al., 2019). There we found that survival processing increased recollection of high-imageability, concrete words, but *decreased* recollection of low-imageability, abstract words, suggesting a strategic allocation of processing resources on an item-by-item basis, rather than on a list basis as in the present study. The apparent discrepancy between the two studies may provide clues as to what features determine a stimulus’s salience, and in turn the amount of resources allocated to processing that stimulus, in the survival context. For example, in the previous study, the mix of concrete and abstract words appears to have created a starker contrast in the survival context than in the moving context, leading to prioritization of concrete words at the expense of abstract words during survival processing. Thus, the potential for functional usefulness, which is presumably higher for concrete words, likely determined a word’s salience to a greater extent in the survival context (Bell et al., 2015; Röer et al., 2013). In the present study, all words were high in imageability and concreteness, and therefore all words presumably had a relatively high potential for usefulness, leading to the homogeneously high allocation of resources in the survival group. In contrast, in the moving group, other possible forms of salience, such as the initial congruence, strangeness, or valence of a word, appear to have been more important, thus creating word-to-word variability in salience and resource allocation. It therefore could be fruitful for future work to explore the relationship between survival processing and different forms of stimulus salience, allowing for a better understanding of how fitness-relevance can influence the allocation of cognitive resources.

### Effect of survival processing on elaborative encoding: evidence from the frontal slow wave

If the survival processing effect is due to richer elaboration during encoding, the frontal slow wave should be enhanced during survival processing. We found that the frontal slow wave began to diverge between the groups approximately 800 ms after word onset and remained more positive in the survival group for the remainder of the 3-second segment. In light of the absence of a group difference in earlier sensory and perceptual ERP components (see Fig. 3 and Footnote 1), and the absence of overall group differences in P300 amplitude, the increased positivity during the frontal slow wave time window provides direct evidence that survival processing selectively up-regulates relatively active, higher-level aspects of encoding. More specifically, previous evidence for an association of the frontal slow wave with elaborative encoding (Johnson, 1995) supports the view that survival processing enhances elaboration (Kroneisen & Erdfelder, 2011). Taken together, the results from the P300 and the frontal slow wave time windows suggest that survival processing reduces the importance of relatively low-level semantic categorization, and increases the importance of later, more active memory encoding processes.

According to our final hypothesis, an increase in elaborative encoding would also be expected to lead to a larger frontal slow wave SME in the survival group. However, we found that word-by-word variation in the amplitude of the frontal slow wave did not predict subsequent memory success in either group, as reflected in a lack of an SME in this time window. This result pattern differs from several studies that have found a frontal slow wave SME elicited by individual words in an incidental encoding paradigm (Kamp et al., 2019; Otten, Quayle, & Puvaneswaran, 2010; Otten & Rugg, 2001; Otten, Sveen, & Quayle, 2007; Paller, McCarthy, & Wood, 1988). One explanation for the absence of a frontal SME in the present study could be that survival processing increased elaboration, reflected in the frontal group difference, but that this elaboration was not related to memory encoding. However, the result pattern is consistent with work that has demonstrated variation in the amplitude of the frontal slow wave unfolding over the entire course of a word list, rather than elicited by individual words, which predicted overall recall rates for the list (Kamp et al., 2016). Therefore, a more likely explanation is that survival processing increased elaboration generally for all words (Craik & Tulving, 1975), rather than selectively for specific words, so the sheer amount of elaboration for a given word was not key to its subsequent retrieval. Instead, the distinctiveness of the retrieval cues (i.e., the ideas about possible object functions) generated during the generally enhanced elaboration may have determined whether a word was later remembered, as suggested by the richness of encoding hypothesis (Bell et al., 2015; Kroneisen & Erdfelder, 2011; Röer et al., 2013), and consistent with our previous work showing increased recollection of episodic details during memory retrieval (Forester et al., 2019). For example, participants in the survival group may have elaborated to a similarly high degree on the words “broom” and “bag,” but if the retrieval cues attached to “broom” were more varied (e.g., cues related to sweeping, defense from a predator, and reaching fruit from a high tree branch) than for “bag” (e.g., carrying tools, carrying weapons, and carrying food), then “broom” would more likely be retrieved and bring to mind more episodic detail during retrieval. This explanation is further supported by oscillatory EEG findings, which showed increased long range phase synchrony associated with encoding in the survival scenario, reflecting widespread cortical communication and suggesting elaboration across multiple domains during survival processing (Fellner et al., 2013).

Although the frontal slow wave difference between groups supports the view that survival processing increases elaboration, it leaves open what *type* of elaboration it influences. Reaching a conclusion in this regard is difficult, because previous evidence is not clear on whether and to what extent the frontal slow wave reflects item-specific elaboration, item-to-item associations, or a combination of these (Kamp et al., 2017). Whereas the richness of encoding hypothesis assumes that survival processing enhances item-specific elaboration, others have proposed that the effect is due to the combination of item-specific and item-to-item (relational) elaboration (Burns, Burns, & Hwang, 2011). Although our ERP findings cannot arbitrate between these two views directly, note that Burns, Hart, Griffith, and Burns (2013) later reported evidence showing that the difference between survival and moving processing is associated with an item-specific processing difference only, not with a difference in relational processing. Also, it is important to keep in mind that our heterogeneous list structure did not provide much opportunity for relational processing of item-to-item associations. It therefore seems more likely that our frontal slow wave reflects item-specific elaboration, rather than item-to-item associations, induced by survival relevance processing.

Another functional view on the frontal slow wave suggests that it reflects the integration of an unexpected but plausible word into its context by suppressing the activation of more expected words (Van Petten & Luka, 2012, for a review). Specifically, a more positive going frontal slow wave is elicited by words that are untypical for, but still congruent with, a given semantic context, relative to both typical words *and* untypical incongruent words, as words from neither of the latter categories necessitate a process of contextual integration. This frontal slow wave effect is thought to reflect the suppression of information in memory that is semantically related to the context, in order to better integrate the untypical or unexpected word into the context (DeLong, Quante, & Kutas, 2014; Höltje, Lubahn, & Mecklinger, 2019). Applied to our results, this view on the functional significance of the frontal slow wave would suggest that the survival context increases the proportion of initially semantically unexpected words that are nevertheless evaluated as plausibly relevant compared with control scenarios. For example, the word “fork” may have a similar (low) degree of semantic relatedness to the survival and moving scenarios. However, a fork may have many potential untypical functions in the survival context, such as serving as a weapon for hunting and defense, or as a tool for gardening, thus encouraging a process of semantic integration in the survival group only (see also Nairne, Coverdale, & Pandeirada, 2019, Exp. 4). Future research is necessary to establish the functional equivalence of the slow waves observed in these different paradigms, and this research could lead to novel insights into the survival processing effect as well as the process of integrating unexpected information into a semantic context.

### Behavioral results: additional findings

The behavioral enhancement of recall due to survival processing was characterized not only by increased true recall, but also by reduced false-memory intrusions. This is inconsistent with prior studies that have reported *increases* in false memory associated with survival processing (Otgaar & Smeets, 2010; Howe & Derbish, 2010), which have been interpreted such that schema or gist processing underlies the survival processing effect. Parker, Dagnall, and Abelson (2018) recently found that survival processing increased false memory for lists of highly associated words (i.e., Deese-Roediger-McDermott word lists), whereas it decreased false memory for words with relatively weaker associations (i.e., taxonomic category lists). Thus, one explanation for the decreased false memory in the present study could be that the strength of semantic associations within our word list was in fact relatively low. Not only were words not selected for their interrelatedness, but it also is feasible that the relatively large number of words in the present study (which were necessary in order to gain a sufficiently high signal-to-noise ratio for the ERP analyses) lead to an especially weak overall cohesion between them. Note, however, that we observed a clear survival processing advantage despite the reverse effect on intrusions. Thus, although understanding the relationship between survival processing and false memory requires future work, the present findings speak against a crucial role for gist or schema processing as underlying the survival processing enhancement effect, or at least against the use of false-alarm rates as evidence for gist processing when word lists are not semantically connected.

## Conclusions

Encoding-related ERP activity indicates that survival processing leads to a shift away from lower level encoding processes to more active and elaborative forms of encoding. The survival processing effect does not appear to be due to a general increase in motivation or arousal, nor does it depend on the initial salience of words within the survival context. Instead, survival processing likely enhances elaboration for any stimulus that offers the potential for contextual elaboration, such as the highly imageable words used in the present study. In summary, these findings are consistent with a richness of encoding account of the survival processing effect.

## Footnotes

1 A reviewer noted that differences in motivation could have influenced ERP activity outside of the P300 time window. We therefore conducted supplementary analyses on the ERP amplitudes of the N1 (75–125 ms) and P2 (150–250 ms) components (Figure 3), which are sensitive to attentional resource allocation. No significant effects involving Scenario were found (all *p*-values > 0.474), providing further evidence that motivation did not vary between the groups and, furthermore, that the effects found within the P300 and slow wave time windows were not due to more global group differences.
